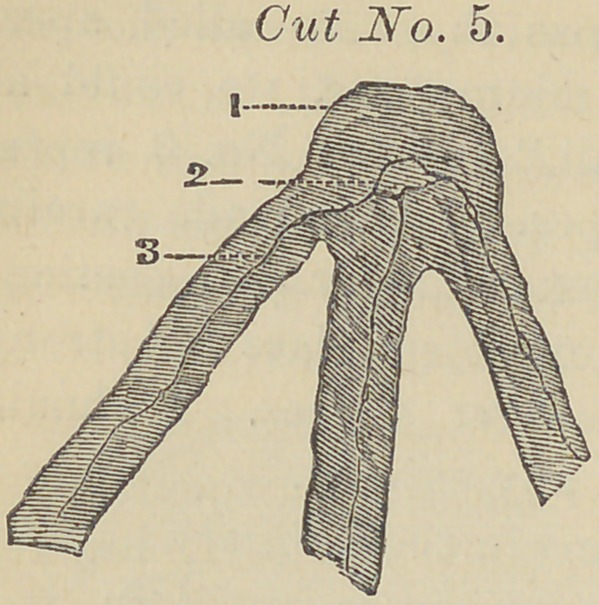# The Dentine Cellular and Fibrous

**Published:** 1872-10

**Authors:** Geo. B. Harriman


### Dental Register.

VOL. XXVI.] OCTOBER, 1872. No. 10.]

### COMMUNICATIONS.

### The Dentine Cellular and Fibrous.

PROF. GEO. B. HARRIMAN.

As has been stated in previous articles, erroneous terms, are employed by writers when considering the structure of the teeth. Because such terms lead to dangerous practice they demand further attention.

Year after year we read of "tubes," "tubuli," "prisms," "hollow pipes," etc. I have for a long period subjected teeth recently extracted as well as others that had been extracted for years to a variety of examinations and experiment. I have taken photographs of a very beautifully-prepared section, sent to me by T. W. Clements, D. D,. Pembroke, Me. The tooth had been extracted five years at least before the section was made, and it represents the cells and fibres most perfectly, and demonstrates beyond a doubt that the "soft solid substances has not dried up." I have, in company with other dentists, made these examinations with one-fiftieth immersion object-glass and achromatic prism for a condenser for illumination, which magnifies five and ten-thousand diameters without the semblance or indication of any "holes or hollow columns or tubuli." All that such examinations show are cells and fibres, which extend from the pulp cavity to the junction of the dentine, with the enamel and cementum, where they anastomose, in many instances two, three, and sometimes more joining and penetrating some distance into the enamel as illustrated by the following cut.

*Cut No.* 1.

This cut represents a thin section of a molar tooth, made by sawing a thin slab and polishing it till it became transparent, and is magnified seven hundred diameters. Figure (1) points to the enamel; figure (2) shows dark thread-like lines, which are nothing more or less than *real fibres*, some of which have penetrated a short distance into the enamel. Figure (3) represent the dentine.

The dark lines or fibres recognized in the cut are what are demonstrated by Prof. McQuillen and some others as "tubuli."

If we glue a portion of the same tooth in a lathe and make a thin turning, and dissolve the calcareous salts or dentine away, we find a soft solid substance or cells closely united into fibres, and the fibres bifurcate and join similar to cut No (1) in this article.

*Cut No.* 2.

Figure (1) in this cut points to the junction of fibres and is magnified four hundred times, thereby showing the swelling of the fibres when the interstitial substance is dissolved.

Were there no soft solid tissues in the dentine, and if it were composed entirely of a hard mineral substance with minute "tubuli" or cavities and apertures, with smooth hard walls, starting from the pulp cavity and penetrating every portion of the dentine, how very easily could this be demonstrated.

Should we make thin sections across these so-called apertures, with the aid of a high power microscope, we could at once see through these holes "tubuli," (as cut No. 3 represents.) These, however, are not pipes. I have made careful and most thorough examination of exceedingly thin sections of dentine with one-fiftieth immersion object-glass and strong condensed light, and failed to discover spaces. I should most assuredly have seen them had any existed.

Not only have I made examinations in the ordinary manner but have taken photographs of the sections mounted in balsam, one of which are represented in the cut No. 4 below.

*Cut No.* 3.

*Cut No.* 4.

Cut No. (3) and (4) are engraved from photograps of my own taking; No. (3) having been treated with a strong solution of caustic potassa disorganizing the animal substance. In treating very thin sections with strong solution of caustic postassa great care is necessary as well as patience and perseverance. The dentine in the operation is liable to break and crumble, much to the annoyance of the experimentor as I well know, and therefore thus write to bid others be of good courage and persevere for perseverance will finally conquer. Again if we submit a section of the dentine to the chemist in its normal condition for being analized, what will be the result? He tells you its chemical composition is about one third animal matter and two thirds mineral substance. Now comes the pertinent inquiry where is the organic substance if the dentine is tubular with smooth hard walls for the "flow and reflow of fluid," as some who would be accounted adepts in dental knowledge affirm.

In the so-called "tubuli" of the dentine, we not only find cells and fibres of connective tissue, but in many instances nerve fibres and ganglion cells can be traced from the pulp cavity to the junction of the enamel, dentine and cementum.

*Cut No. 5.*

Figure (1) points to the termination of the fibres where they unite.

Figure (2) presents the ganglion cell in which can be seen three small nerve filaments. Figure (3) points to one of the small nerve fibres running from the pulp and constituting a junction or ganglion 2. It will be noticed that the junction of fibres is made at the enamel, and that the calcareous salts cleared away with the acid, and that the section is very highly magnified. With the use of terchloride of gold I am enabled after long and patient toil with very delicate, fine pointed needles, under low magnifying power to dissect out some of these fine filaments, measuring less than the fifty-thousandth of an inch in diameter. By a careful examination with the one-fifty object glass, I am satisfied that it is the axis cylinder without the medullary grey substance.

It is a well-known fact to histological observers that the medulla does not appear until afterward, when nerves become broader and are more easily defined. It is not hence absolutely necessary for a constituent of a sensitive nerve to contain the medullary sheath.

The individuals who do not admit the existence of the axis cylinder regard the white substance not only as the predominating constituent but also as a really active element of the nerve contents. Prof. Cutler of the New Orleans Dental College, regards the whole of the soft solid substances as nerve fibre. By making a chemical examination of the entire substance in the so-called "tubuli" the result is a large amount of the medullary substance, and that there scarcely exists a tissue rich in cells where this substance does not occur in large quantity. Still it is only in the nerve fibres that we observe the peculiarity of the substance as such, whilst in all other cellular elements it is contained in a finely divided state in the interior of the cells, and is only set free as the contents undergo a change, and are subjected to the action of chemical regents. From blood cells, pus corpuscles, epithilium cells of the most versatile glandular parts, from the interior of the spleen and similar organs, unprovided with excretory ducts, this substance can in every case be obtained by extraction. Hence it is manifest that the medulla can not be the constituent in which reposes the function of the nerve as such. This same conclusion is arrived at by physical in vestigations at the present time. Therefore the axis cylinder very generally regarded as the real essential constituent of the nerves whilst in the white ones it can only be isolated separation of the investing medulallary sheath. The *axis cylinder* would hence seem to be the *real electrical substance* and we may certainly admit the hypothesis that the medullary sheath rather serves as an isolating mass, which confines the electricity within the nerve itself and allows its discharge at the non-medullated extremities of the fibres.

I next consider the termination of the fibres, my conclu-clusions are neither sudden nor premeditated. To the subject I have given great attention, especially as they are ob served in one of the tissues. I differ from authorities. I have proof of my theories.

Various forms of termination are given to nerves. Do they end in points terminating in the tissues or cell of the part? or do they end in loops? or are they plexuses? If we take the teeth here, we find the nerve fibres passing through into the dentine towards the enamel and cementum, and in some instances they can be traced entering its substance. As the fibres approach their termination they draw together and unite by anastomosis or rather they enter in a cell or ganglion; but in *no instance do they terminate by free extremity.* This formation somewhat confirms the position that all nerve fibres form a complete circuit for the current of nerve force whether it be motar or sensor. I am confident they do not end in points or filaments. Nerves in the teeth have no ends except as they are continuous with the terminating portion of another nerve fibre or as they terminate in what I will call a *ganglion cell.* This termination of nerve fibres in dentine and enamel, this ganglion or cell termination of nerves in teeth never before demonstrated, now being proved and accepted we have now to seek in some form for the cause of excessive sensation in teeth. Fibres surrounded with such bony encasements, we might well expect would be very susceptible to the slightest disturbance or jar, and especially when the equilibrium of its force was unbalanced.

The termination of motor nerves to muscle is by plexuses ramifying over and between the fibres of the muscles. In no case can they be seen to enter into them, they simply lie upon them and exert their influence by transmission or contact.

Much more might be said in elucidation of the positions taken in this and the preceding articles. It is hoped what has been submitted will excite thoughtfulness and provoke investigation.

## Figures and Tables

**Cut No. 1. f1:**
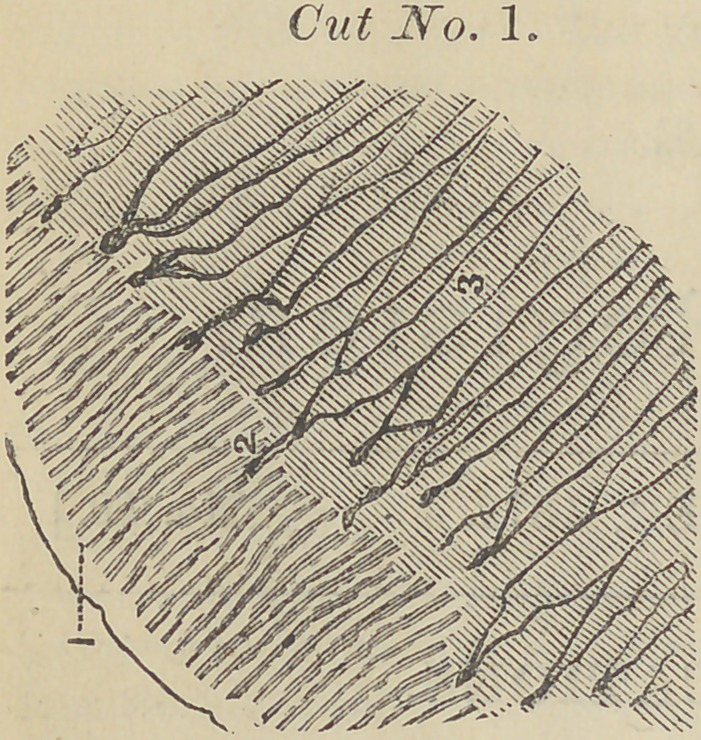


**Cut No. 2. f2:**
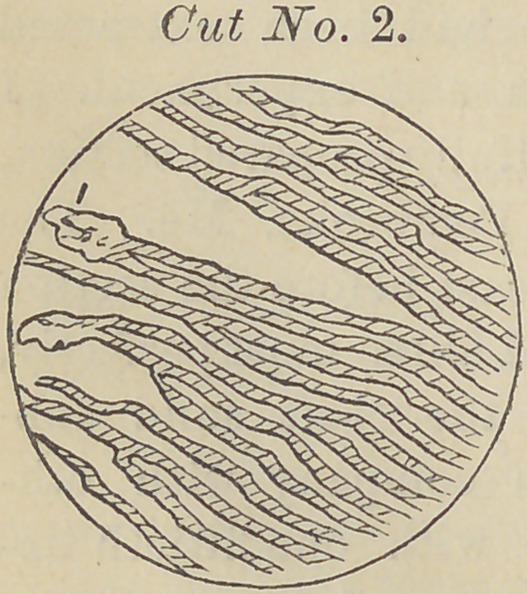


**Cut No. 3. f3:**
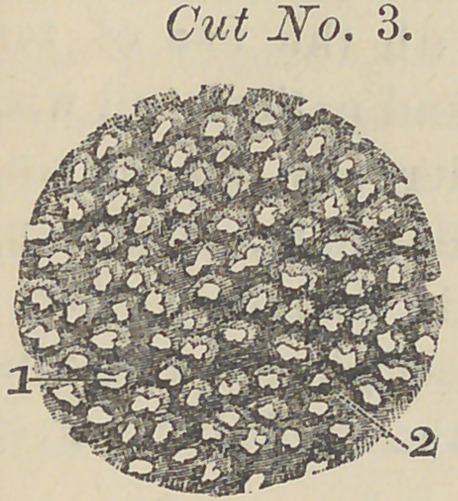


**Cut No. 4. f4:**
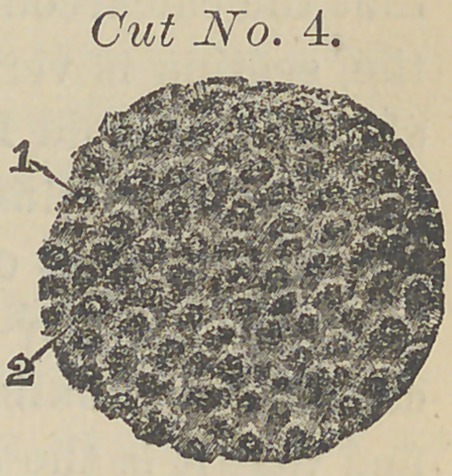


**Cut No. 5. f5:**